# Relationship between lung function and quantitative computed tomographic parameters of airway remodeling, air trapping, and emphysema in patients with asthma and chronic obstructive pulmonary disease: A single-center study

**DOI:** 10.1016/j.jaci.2016.02.001

**Published:** 2016-05

**Authors:** Ruth A. Hartley, Bethan L. Barker, Chris Newby, Mini Pakkal, Simonetta Baldi, Radhika Kajekar, Richard Kay, Marie Laurencin, Richard P. Marshall, Ana R. Sousa, Harsukh Parmar, Salman Siddiqui, Sumit Gupta, Chris E. Brightling

**Affiliations:** a Department of Infection, Inflammation and Immunity and Health Sciences, Institute for Lung Health, University of Leicester, Leicester, United Kingdom; b Radiology Department, Glenfield Hospital, University Hospitals of Leicester NHS Trust, Leicester, United Kingdom; c Department of experimental Medicine, Hoffmann-La Roche, Nutley, NJ; d Novartis, Basel, Switzerland; e GlaxoSmithKline, Stevenage, United Kingdom

**Keywords:** Asthma, chronic obstructive pulmonary disease, airway remodeling, quantitative computed tomography, asthma-COPD overlap syndrome, small airway disease, emphysema, gas trapping, BSA, Body surface area, COPD, Chronic obstructive pulmonary disease, CT, Computed tomography, GINA, Global Initiative for Asthma, ICC, Intraclass correlation, KCO, Transfer coefficient, LA, Lumen area, MLD_E/I_, Mean lung density expiratory/inspiratory ratio, Perc15, Hounsfield units below which 15% of the voxels lie, QCT, Quantitative computed tomography, TA, Total area, WA, Wall area

## Abstract

**Background:**

There is a paucity of studies comparing asthma and chronic obstructive pulmonary disease (COPD) based on thoracic quantitative computed tomographic (QCT) parameters.

**Objectives:**

We sought to compare QCT parameters of airway remodeling, air trapping, and emphysema between asthmatic patients and patients with COPD and explore their relationship with airflow limitation.

**Methods:**

Asthmatic patients (n = 171), patients with COPD (n = 81), and healthy subjects (n = 49) recruited from a single center underwent QCT and clinical characterization.

**Results:**

Proximal airway percentage wall area (%WA) was significantly increased in asthmatic patients (62.5% [SD, 2.2]) and patients with COPD (62.7% [SD, 2.3]) compared with that in healthy control subjects (60.3% [SD, 2.2], *P* < .001). Air trapping measured based on mean lung density expiratory/inspiratory ratio was significantly increased in patients with COPD (mean, 0.922 [SD, 0.037]) and asthmatic patients (mean, 0.852 [SD, 0.061]) compared with that in healthy subjects (mean, 0.816 [SD, 0.066], *P* < .001). Emphysema assessed based on lung density measured by using Hounsfield units below which 15% of the voxels lie (Perc15) was a feature of COPD only (patients with COPD: mean, −964 [SD, 19.62] vs asthmatic patients: mean, −937 [SD, 22.7] and healthy subjects: mean, −937 [SD, 17.1], *P* < .001). Multiple regression analyses showed that the strongest predictor of lung function impairment in asthmatic patients was %WA, whereas in the COPD and asthma subgrouped with postbronchodilator FEV_1_ percent predicted value of less than 80%, it was air trapping. Factor analysis of QCT parameters in asthmatic patients and patients with COPD combined determined 3 components, with %WA, air trapping, and Perc15 values being the highest loading factors. Cluster analysis identified 3 clusters with mild, moderate, or severe lung function impairment with corresponding decreased lung density (Perc15 values) and increased air trapping.

**Conclusions:**

In asthmatic patients and patients with COPD, lung function impairment is strongly associated with air trapping, with a contribution from proximal airway narrowing in asthmatic patients.

Asthma and chronic obstructive pulmonary disease (COPD) cause considerable morbidity and consume substantial health care resources.^1^, ^2^ Both airway diseases are characterized by airflow obstruction, which is typically variable and reversible in asthmatic patients but fixed in patients with COPD.^3^ However, there is overlap between the 2 conditions, particularly between patients with severe asthma and those with COPD, because severe asthma can be characterized by persistent airflow obstruction and some patients with COPD have partially reversible airflow obstruction. Similarly, there is emerging evidence of overlap between asthma and COPD in terms of inflammatory profiles, with the former typically associated with eosinophilic and the latter with neutrophilic inflammation, but both patterns were observed in subgroups of each disease.^3^, ^4^, ^5^

Quantitative computed tomography (QCT) has become an established technique for airway morphometry and lung densitometry in patients with airway disease.^6^, ^7^, ^8^ This approach allows for quantification of proximal airway remodeling by means of assessment of airway lumen and wall geometry, air trapping as an indirect measure of small-airway disease, and emphysema determined by means of lung densitometry. QCT has been applied extensively to patients with COPD. Indeed, a systematic review in 2012 found that both markers of emphysema and peripheral airway measurements correlated with airflow obstruction in patients with COPD.^9^ QCT in patients with COPD is generally accepted as a robust method, especially for quantifying emphysema.^10^ QCT-measured emphysema has been shown to predict mortality^11^ and has been linked to lung function decrease.^12^ QCT in asthmatic patients has demonstrated tremendous heterogeneity in airway remodeling, showing that change in lumen dimension is an important aspect of proximal airway remodeling^8^ and identifying that changes in airway geometry are associated with histologic features of airway remodeling.^13^, ^14^, ^15^ Whether the relationships between lung function and QCT parameters are different in asthmatic patients and patients with COPD is uncertain.

Our hypotheses were as follows: (1) QCT morphometric and densitometric measures of proximal airway remodeling, air trapping, and emphysema are different between asthmatic patients, patients with COPD, and healthy subjects, and (2) in asthmatic patients and patients with COPD, the association between lung function impairment (postbronchodilator FEV_1_ percent predicted) and these QCT morphometric and densitometric measures are distinct. The coprimary QCT outcome variables were as follows: mean airway lumen area (LA)/body surface area (BSA) and percentage wall area (%WA) for proximal airway remodeling; mean lung density expiratory/inspiratory ratio (MLD_E/I_) for air trapping; and Hounsfield units below which 15% of the voxels lie (Perc15) for emphysema.

To test our hypotheses, we undertook a QCT observational study of asthmatic patients and patients with COPD across the spectrum of disease severity and investigated the relationship between lung function and QCT parameters first in each disease and second in QCT-derived clusters of the disease groups combined. Some of the results of this study have been previously reported in the form of an abstract.^16^, ^17^

## Methods

### Subjects

Adults with COPD (n = 81) or asthma (n = 171) and healthy control subjects (n = 49) were recruited at a single center, Glenfield Hospital, Leicester, United Kingdom. Patients with COPD and asthmatic patients were recruited from respiratory outpatient clinics, and healthy control subjects were recruited through posters and advertisements placed in public areas, including outpatient clinics in the hospital, support group meetings, and leisure centers. Patients with COPD and asthmatic patients fulfilled diagnostic criteria per Global Initiative for Chronic Obstructive Lung Disease and Global Initiative for Asthma guidelines, respectively.^18^, ^19^ Patients with COPD had a greater than 10 pack year smoking history and were more than 40 years old. Twenty-nine healthy control subjects and 60 asthmatic patients participated in previous studies.^7^, ^8^ The study was approved by the Leicestershire Ethics Committee, and patients provided written informed consent.

It was ensured that all subjects with airway disease at the time of study visits were free from an exacerbation requiring systemic corticosteroids, antibiotics, or both for at least 6 weeks. All subjects underwent extensive clinical characterization, including thoracic computed tomography (CT), lung function tests, spirometry, full blood count, sputum analysis, and health status questionnaires. The St Georges Respiratory Questionnaire for patients with COPD and the Asthma Quality of Life Questionnaire and Asthma Control Questionnaire for asthmatic patients were used.

### CT

Volumetric whole lung scans were obtained by using a Siemens Sensation 16 scanner at a single center at full inspiration (near total lung capacity) and at the end of expiration (near functional residual capacity). Details of CT acquisition and quantitative airway morphometry and lung densitometry are provided in the Methods section in this article's Online Repository at www.jacionline.org. All scans were analyzed by a single observer (RH) using the semiautomated software Apollo (VIDA Diagnostics, Coralville, Iowa), and various QCT parameters were obtained. Scans from 76 subjects were analyzed by 2 observers (RH and SG) for assessment of interobserver repeatability (see the Methods section in this article's Online Repository).

### Statistical analysis

Statistical analyses were performed with IBM SPSS Statistics software for Windows (version 20.0; IBM, Armonk, NY) and GraphPad Prism software for Windows (version 6; GraphPad Software, San Diego, Calif). *A priori* subject stratification determined based on postbronchodilator FEV_1_ percent predicted measurement was performed. Nonparametric and parametric data were presented as medians (interquartile ranges) or means (SDs), respectively. Comparisons across groups were analyzed by using parametric and nonparametric ANOVA with *post hoc* testing for pairwise comparisons. Pairwise comparisons were made by using *t* tests or Mann-Whitney tests, as appropriate. Statistical significance was reached if the *P* value was less than .05. Factor and cluster analysis were carried out with IBM SPSS Statistics software for Windows (version 20.0). The Kaiser criterion was used to select the number of the factors, and Ward hierarchical clustering was used to determine the number of clusters (k). Cluster membership was derived by using k-means clustering (see the Methods section in this article's Online Repository for further details).

## Results

### Clinical characteristics

The baseline demographics and clinical characteristics of asthmatic patients, patients with COPD, and healthy subjects are shown in Table I. Patients with COPD were older and had a greater smoking pack year history, poorer lung function (airflow limitation defined as postbronchodilator FEV_1_ percent predicted <80% or airflow obstruction defined as postbronchodilator FEV_1_/forced vital capacity ratio <70%), and higher neutrophilic airway inflammation compared with that in asthmatic patients. Asthmatic patients had higher eosinophilic airway inflammation compared with the other 2 groups. Body mass index of asthmatic patients was greater than that of patients with COPD. Poorer lung function was also demonstrated in asthmatic patients compared with that seen in healthy control subjects.

### QCT parameters: Comparison between asthmatic patients, patients with COPD, and healthy subjects

**Fig E1:**
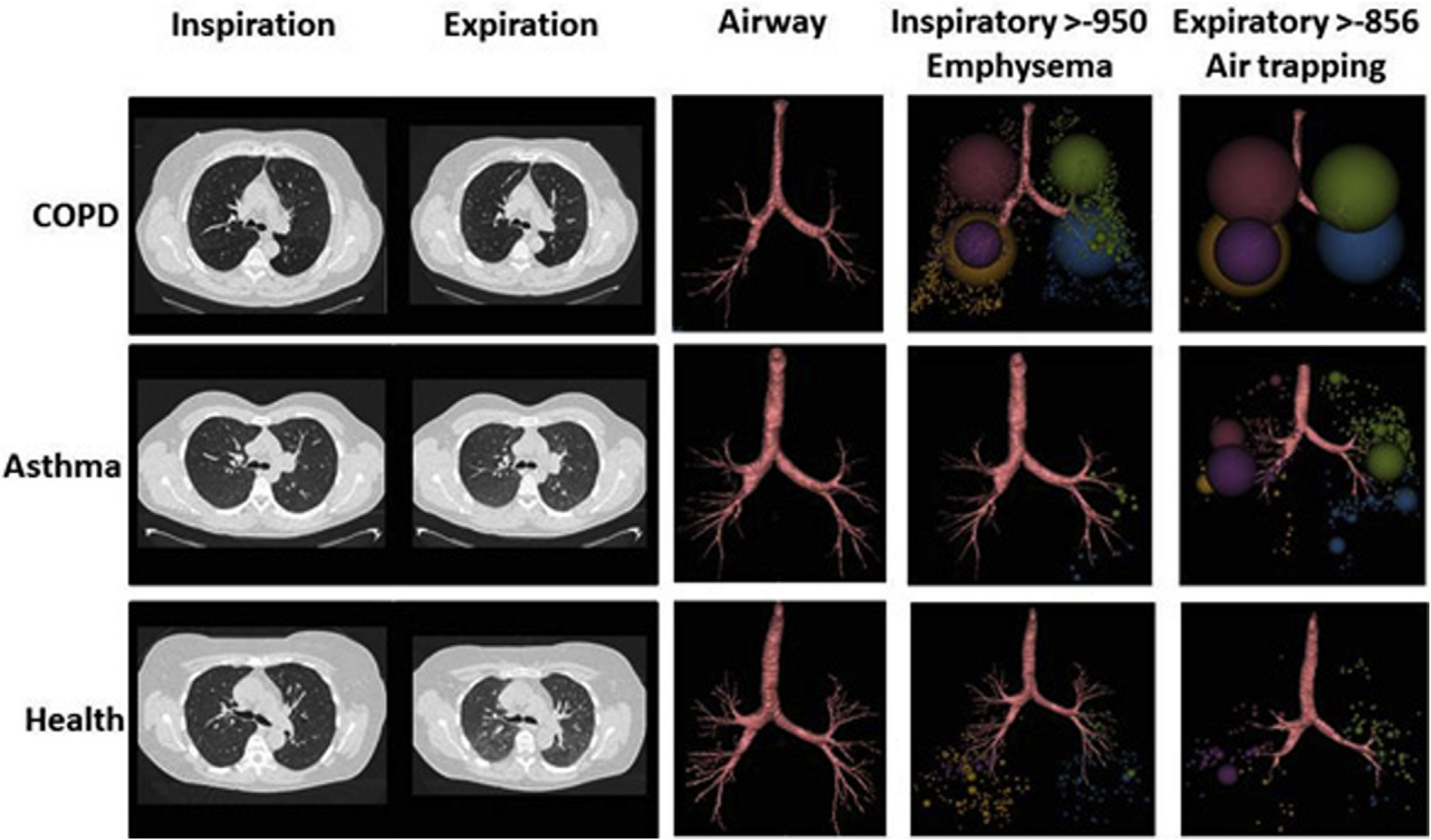
Visual representation of QCT parameters with CT inspiratory and expiratory axial slices *(first 2 columns)* illustrates qualitative differences of increased inspiratory volume, emphysema, and air trapping in patients with disease versus healthy control subjects. *Column 3* shows airways grown by the postprocessing software, which are reduced in patients with disease. The *fourth column* shows low attenuating clusters (LACs) of less than −950 HU in inspiration, representing areas of emphysema, and the *final column* shows the LACs of less than −856 in expiration, representing areas of air trapping.

Examples of CT images for asthmatic patients, patients with COPD, and healthy control subjects are as shown in Fig E1 in this article's Online Repository at www.jacionline.org. Airway morphometry and lung densitometry for asthmatic patients, patients with COPD, and healthy subjects are summarized in Table II. Segmental airway morphometry is shown in Tables E1 and E2 in this article's Online Repository at www.jacionline.org. Interobserver repeatability for QCT parameters was good to excellent (see the Methods section in this article's Online Repository).

**Fig 1:**
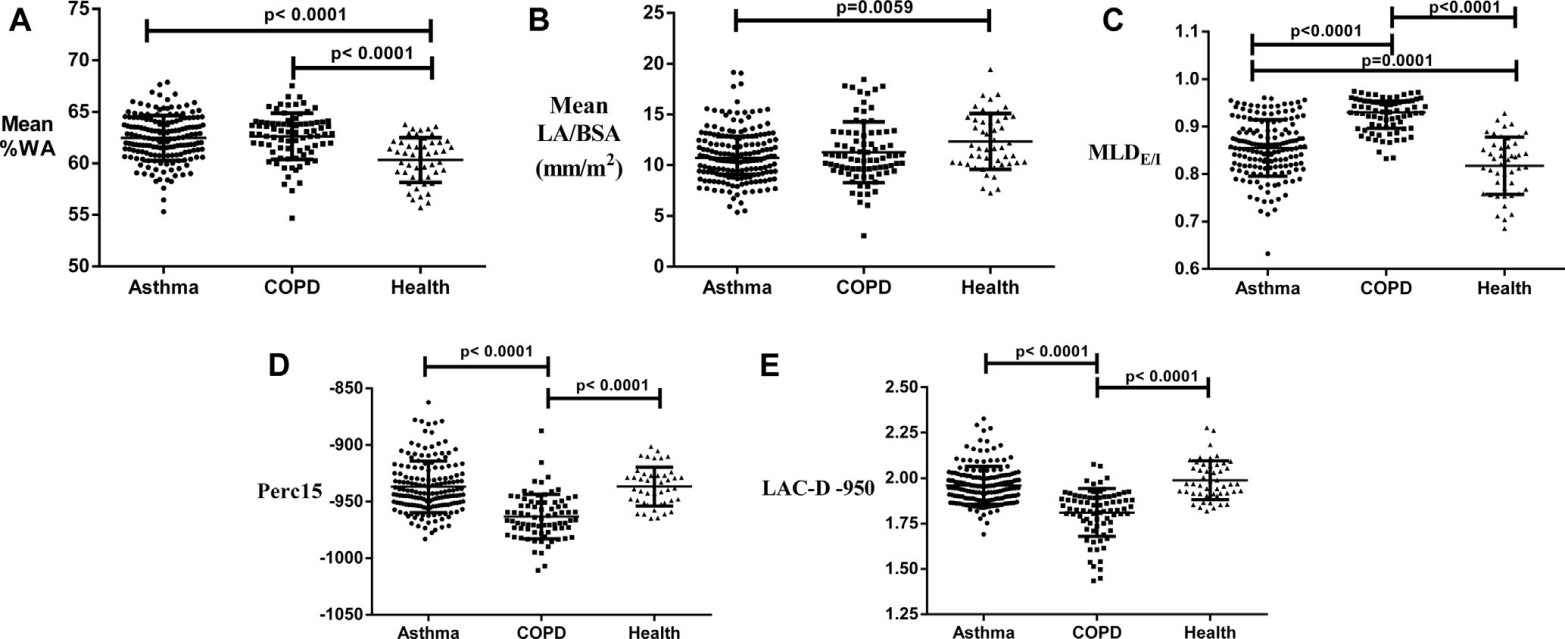
Dot plots of airway morphometric and densitometric QCT parameters for all asthmatic patients, patients with COPD, and healthy control subjects: **A,** mean %WA; **B,** mean LA/BSA; **C,** MLD_E/I_; **D,** densitometry (Perc15); and **E,** fractal index (LAC-D−950).

Mean WA/BSA values were not significantly different among the 3 groups. However, the mean %WA was increased in both asthmatic patients and patients with COPD compared with that in healthy control subjects, with mean LA/BSA values being significantly smaller in asthmatic patients. The mean LA/BSA value was less in patients with COPD compared with that in healthy control subjects, although it did not reach statistical significance (Fig 1, *A* and *B*, and Table II). MLD_E/I_ was increased in both asthmatic patients and patients with COPD compared with that in healthy control subjects, with the highest values seen in patients with COPD (Fig 1, *C*, and Table II). Perc15 values were decreased only in patients with COPD, with comparable values in asthmatic patients and healthy subjects (Fig 1, *D*, and Table II). Low-attenuation clusters of less than −950 HU fractal dimension (LAC-D−950) value were significantly decreased in patients with COPD (Fig 1, *E*). WA of the theoretical airway with an internal perimeter of 10 mm and %WA of a theoretical airway with an external perimeter of 20 mm values were increased in both asthmatic patients and patients with COPD compared with those in healthy control subjects (Table II). Age-adjusted comparison of the coprimary QCT parameters between asthmatic patients, patients with COPD, and healthy subjects was performed because the mean age of patients with COPD was higher compared with that of other groups, and all of the comparisons (1-way ANOVA) remained statistically significant (*P* < .001).

### Univariate analysis to explore the structure-function relationship in asthmatic patients and patients with COPD

**Fig 2:**
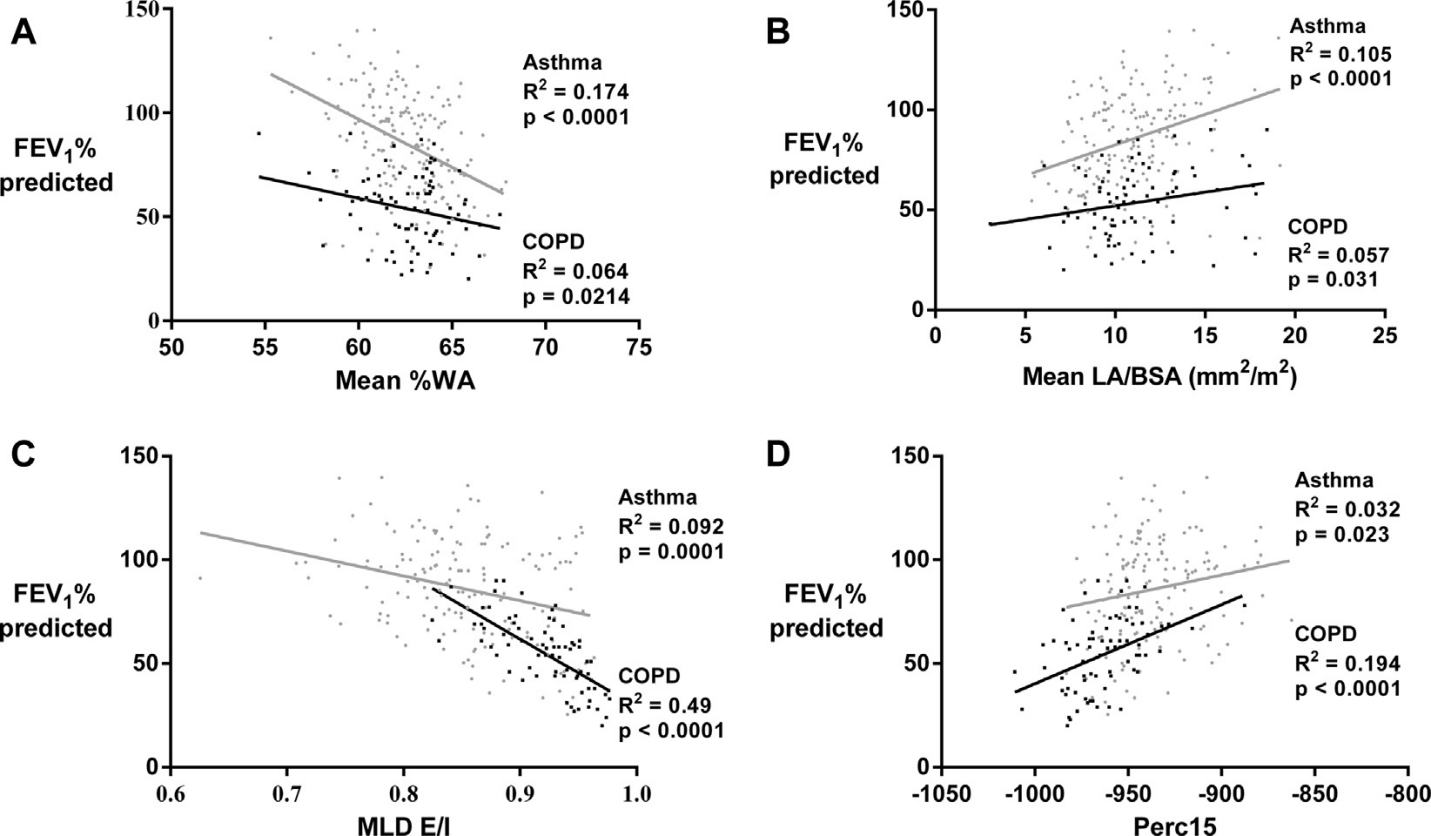
Scatter plot and linear regression of asthmatic patients *(gray circles)* and patients with COPD *(black squares)* showing the relationship between FEV_1_ percent predicted values and the QCT morphometric and densitometric measures: **A,** mean %WA; **B,** mean LA/BSA; **C,** MLD_E/I_; and **D,** densitometry (Perc15).

Correlations between the QCT indices and clinical or physiologic parameters are shown in Table III and Table E3 in this article's Online Repository at www.jacionline.org. Moderate-to-good correlations were observed between QCT parameters and lung physiology indices. Perc15 values were strongly correlated with transfer coefficient (KCO) percent predicted values in patients with COPD and MLD_E/I_, with residual volume/total lung capacity (as a percentage) in all 3 groups. Airflow obstruction was most strongly associated with Perc15 and MLD_E/I_ values, with a weaker association with %WA and LA/BSA values in asthmatic patients and patients with COPD (Table III). Airflow limitation in asthmatic patients was strongly correlated with mean %WA and weakly with MLD_E/I_ and Perc15 values. In contrast, airflow limitation in patients with COPD was most strongly associated with MLD_E/I_ and, to a lesser extent, Perc15 and %WA values (Fig 2 and Table III). Sputum neutrophil counts showed positive correlations with mean %WA values in asthmatic patients, and sputum eosinophil counts were inversely correlated with mean %WA values in patients with COPD. Correlations were also observed between (1) airway narrowing and asthma control and (2) between MLD_E/I_ and COPD-related quality of life (see Table E3).

### Multiple regression analysis to explore structure-function relationships in asthmatic patients and patients with COPD

Multiple linear regression analysis in asthmatic patients showed that mean %WA, MLD_E/I_, and Perc15 values made a statistically significant contribution to the regression model for prediction of postbronchodilator FEV_1_ percent predicted, with mean %WA values making the strongest unique contribution. Multiple linear regression analysis in patients with COPD showed that MLD_E/I_ and mean %WA values made a statistically significant contribution to the regression model for prediction of postbronchodilator FEV_1_ percent predicted, with MLD_E/I_ making the strongest unique contribution (Table IV).

### Univariate and multiple regression analysis to explore structure-function relationships in asthmatic patients and patients with COPD with airflow limitation

**Fig E2:**
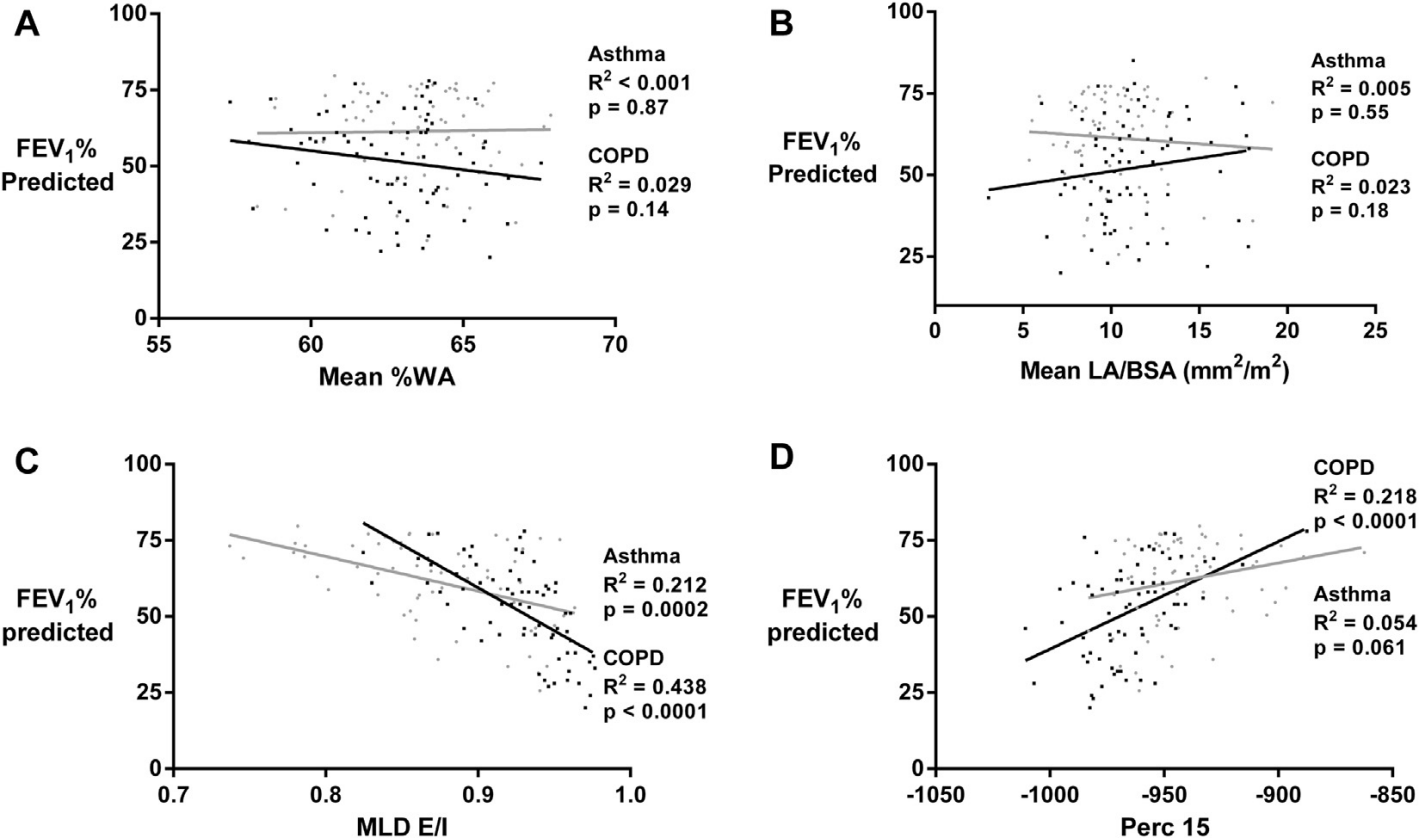
Scatter plot and linear regression of subjects with an FEV_1_ percent predicted value of less than 80% with asthma *(gray circles)* and COPD *(black squares)*, showing the relationship between FEV_1_ percent predicted and QCT morphometric and densitometric measures: **A,** mean %WA; **B,** mean LA/BSA; **C,** MLD_E/I_; and **D,** densitometry (Perc15).

A subset of asthmatic patients and patients with COPD with postbronchodilator FEV_1_ percent predicted values of less than 80% were assessed for correlations between QCT and lung physiology parameters (Fig E2 and Table E4 in this article's Online Repository at www.jacionline.org). Correlations between KCO percent predicted or residual volume/total lung capacity (as a percentage) and Perc15 or MLD_E/I_ values were stronger compared with previous analysis of unselected patients (see Table E4). Postbronchodilator FEV_1_ percent predicted values showed correlations with MLD_E/I_ in asthmatic patients and with both MLD_E/I_ and Perc15 values in patients with COPD. Multiple linear regression analysis demonstrated that in this subset of patients with COPD, MLD_E/I_ made the strongest unique contribution to the regression model for prediction of postbronchodilator FEV_1_ percent predicted (see Table E5 in this article's Online Repository at www.jacionline.org). Multiple regression analysis was not performed in asthmatic patients because univariate analysis only showed correlation between postbronchodilator FEV_1_ percent predicted and MLD_E/I_.

### Asthma and COPD subgroup analysis

**Fig 3:**
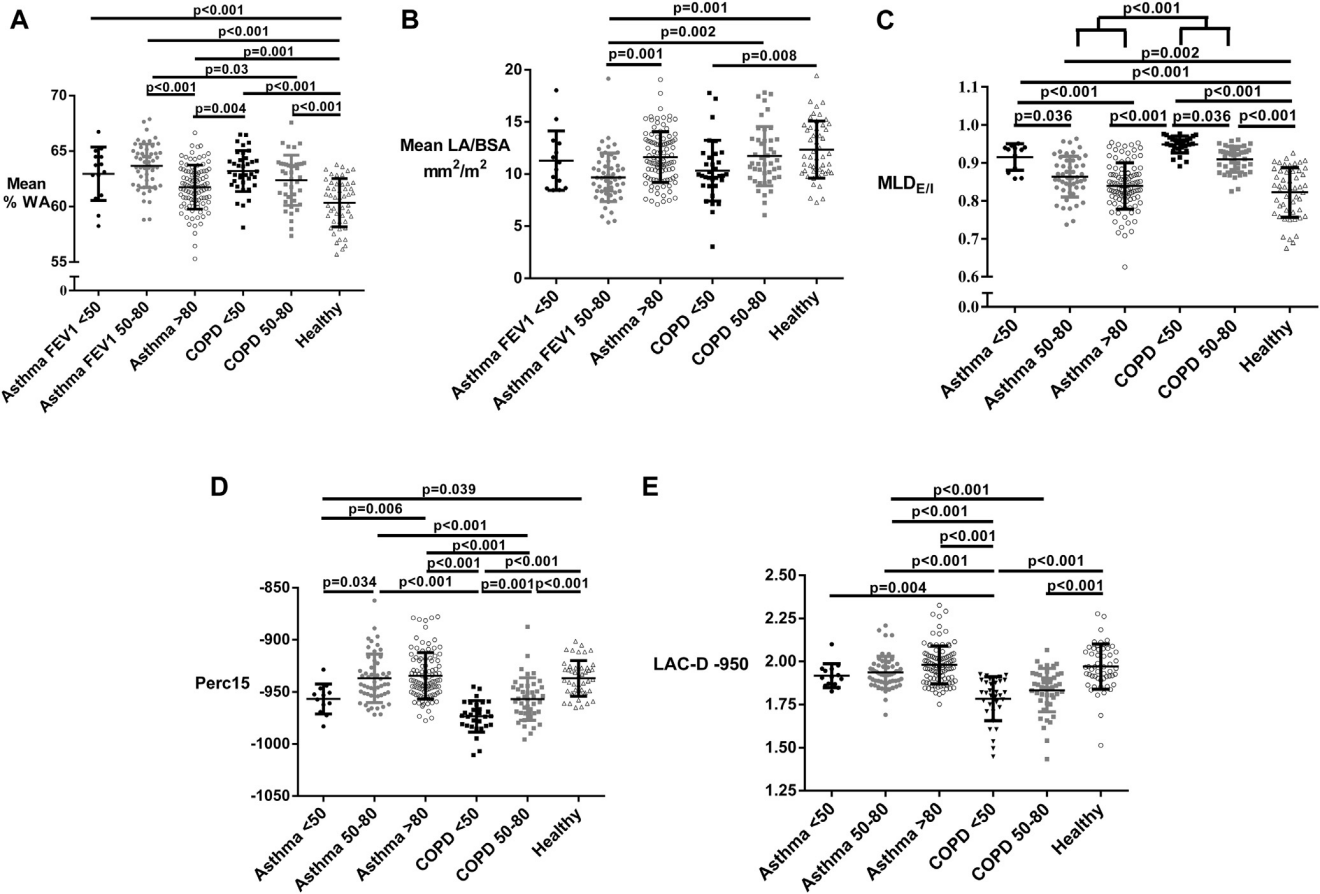
Dot plots of airway morphometric and densitometric QCT parameters for asthmatic patients (*black circles*, FEV_1_ percent predicted <50%; *gray circles*, 50% to 80%; *open circles*, >80%), patients with COPD (*black squares*, FEV_1_ percent predicted <50%; *gray squares*, 50% to 80%), and healthy control subjects *(open triangles)*: **A,** mean %WA; **B,** mean LA/BSA; **C,** MLD_E/I_; **D,** densitometry (Perc15); and **E,** fractal index (LAC-D−950).

We stratified asthmatic patients and patients with COPD into 3 subgroups each based on postbronchodilator FEV_1_ percent predicted: (1) greater than 80% (asthma, n = 101; COPD, n = 5), (2) 50% to 80% (asthma, n = 56; COPD, n = 43), and (3) less than 50% (asthma, n = 14; COPD, n = 34). Because only 5 patients with COPD had a postbronchodilator FEV_1_ percent predicted value of greater than 80%, they were excluded from further analyses. The asthmatic patients with postbronchodilator FEV_1_ percent predicted values of greater than 80% compared with healthy control subjects have significantly greater mean %WA values, with no significant difference in MLD_E/I_ or Perc15 values (Fig 3, *A-D*). In the asthma subgroup with postbronchodilator FEV_1_ percent predicted values of 50% to 80%, mean %WA values were greater and LA/BSA values were lower compared with those in the subgroup with postbronchodilator FEV_1_ percent predicted values of greater than 80% (Fig 3, *A* and *B*). The asthma subgroup with FEV_1_ percent predicted values of less than 50% did not show a significant difference in airway morphometry compared with the other asthma subgroups. In patients with COPD, mean %WA and LA/BSA values were not significantly different between the subgroups with postbronchodilator FEV_1_ percent predicted values of 50% to 80% versus less than 50%. In subgroups with postbronchodilator FEV_1_ percent predicted values of 50% to 80%, asthmatic patients have greater mean %WA and smaller LA/BSA values compared with those in patients with COPD (Fig 3, *A* and *B*).

In both asthmatic patients and patients with COPD, subgroups with lower postbronchodilator FEV_1_ percent predicted values had higher MLD_E/I_ and lower Perc15 values (Fig 3, *C* and *D*). The asthma and COPD subgroups with a similar degree of lung function impairment showed no significant difference in MLD_E/I_ values (Fig 3, *C*). Patients with COPD with postbronchodilator FEV_1_ percent predicted values of 50% to 80% showed decreased Perc15 values compared with those in asthmatic patients with a similar degree of lung function impairment (Fig 3, *D*). In subgroups with postbronchodilator FEV_1_ percent predicted values of less than 50%, patients with COPD and asthmatic patients showed no significant difference in Perc15 values (Fig 3, *D*), but the LAC-D−950 value was significantly decreased in patients with COPD (Fig 3, *E*).

### Unbiased phenotyping of patients with airway disease (asthma and COPD) by using factor analysis of QCT parameters

**Fig E3:**
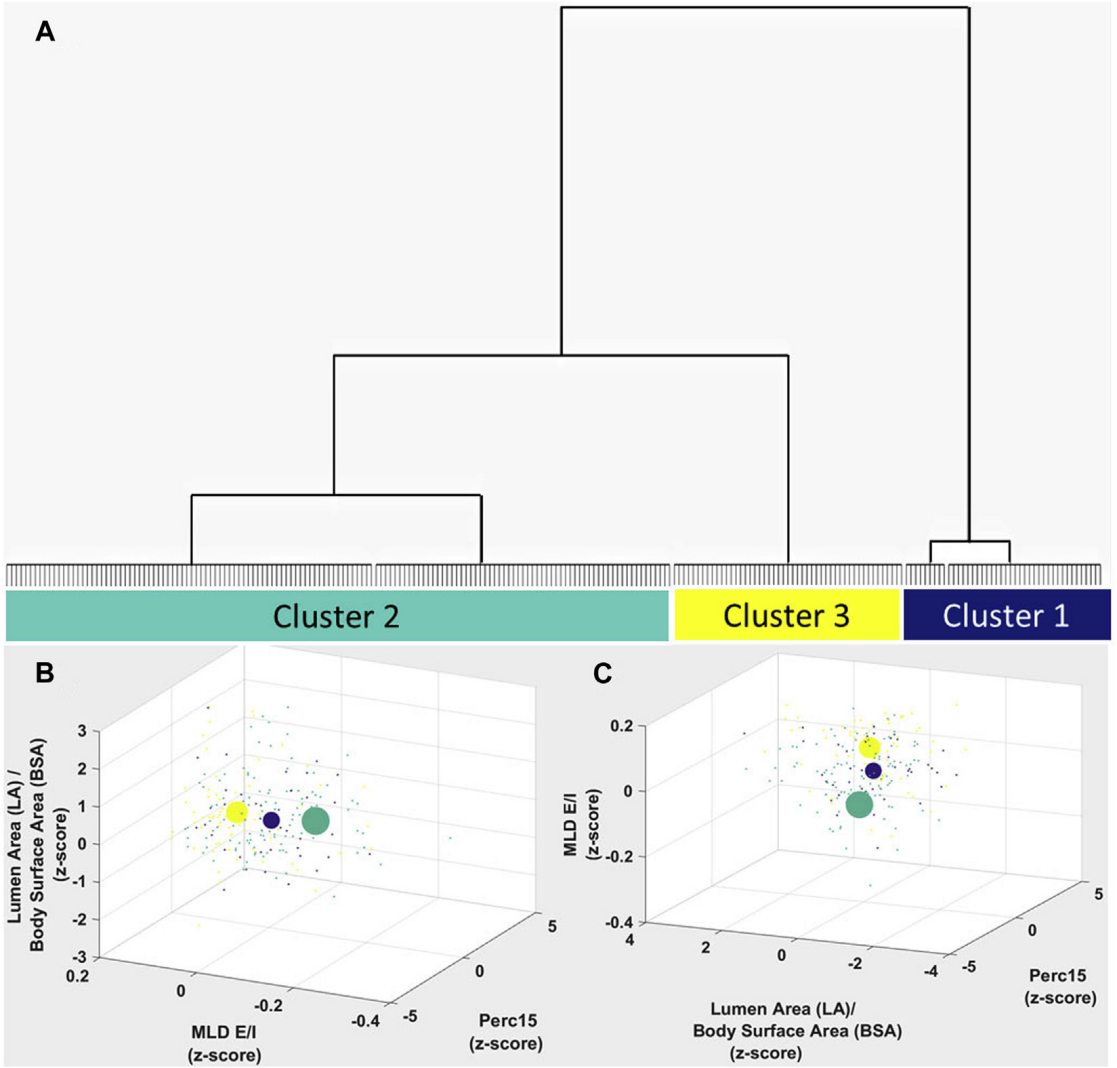
**A,** Dendrogram to which the number of clusters was determined (k = 3). **B** and **C,** Two representations of the clusters on z scores of 3 QCT variables: MLD_E/I_, Perc15, and LA/BSA. *Small dots* represent individual patients, and *large spheres* represent sample sizes of clusters centered on their multivariate cluster means.

We undertook a *de novo* factor analysis of QCT parameters in asthmatic patients or patients with COPD, which revealed the 3 components with the strongest loading variables as mean LA/BSA, Perc15, and MLD_E/I_ values (see Table E6 in this article's Online Repository at www.jacionline.org). A cluster analysis using these 3 highest loading variables revealed 3 clusters (see Fig E3 and Table E7 in this article's Online Repository at www.jacionline.org). The 3 clusters had mild (asthma, n = 40; COPD, n = 2), moderate (asthma, n = 94; COPD, n = 24), and severe (asthma, n = 25; COPD, n = 47) lung function impairment, respectively, with decreased Perc15 and increased MLD_E/I_ values, which are a particular feature of cluster 3.

## Discussion

We describe the airway morphometry and lung densitometry of asthmatic patients and patients with COPD with reference to healthy control subjects and their relationship to lung function. We found that proximal airway remodeling and air trapping were features of both asthma and COPD. Airway WA, expressed as a percentage of total area (%WA), was increased in patients with either disease. Air trapping in patients with COPD was more severe compared with that in asthmatic patients. Emphysema was only seen in patients with COPD, with Perc15 values being significantly lower compared with those in other groups. Comparable Perc15 values between asthmatic patients and healthy subjects confirm the absence of emphysema in asthmatic patients. Assessment of structure-function relationships revealed a significant contribution of proximal airway remodeling, which was represented by the percentage of WA and air trapping, as represented by MLD_E/I_, in the prediction of airflow limitation in asthmatic patients. In contrast, similar assessment in patients with COPD showed that only QCT-determined air trapping and emphysema contributed to airflow limitation. Both disease groups, when further stratified by the degree of lung function impairment, showed that in the subgroup with postbronchodilator FEV_1_ percent predicted values of less than 80%, air trapping remained a significant predictor of lung function impairment. Proximal airway remodeling in this group of subjects did not contribute to prediction of airflow limitation.

With asthma and COPD combined in a factor and cluster analysis, the findings were consistent with our *a priori* stratification. Factor analysis revealed 3 components, with the highest loading factors being measures of proximal airway narrowing, air trapping, and emphysema, and cluster analysis demonstrated 3 clusters that could be distinguished by their degree of airflow obstruction.

Changes in proximal airway geometry in patients with COPD are common, and our findings of increased mean segmental %WA values compared with those in control subjects was consistent with previous studies.^20^ This is consistent with proximal airway remodeling in asthmatic patients in the current and previous studies.^8^, ^21^ Diaz et al^22^ have also demonstrated proximal airway lumen narrowing in patients with mild COPD. No significant difference was seen in proximal airway remodeling between asthmatic patients and patients with COPD, which is consistent with previous literature.^23^ Conversely, other studies report significantly greater proximal airway remodeling in asthmatic patients compared with that seen in patients with COPD.^24^, ^25^ In our study the asthma subgroup with postbronchodilator FEV_1_ percent predicted values of 50% to 80% have greater mean %WA and smaller LA/BSA values compared with those in patients with COPD, with a similar degree of airflow limitation. Moreover, among airway disease subgroups with postbronchodilator FEV_1_ percent predicted values of less than 50% when compared with those in healthy control subjects, proximal airway lumen narrowing was seen in patients with COPD but not in asthmatic patients. These findings highlight the heterogeneity of airway disease and the importance of multilevel disease phenotyping and suggest that proximal lumen dimensions in asthmatic patients with severe airflow impairment might become relatively dilated, perhaps to compensate for progressive small-airway disease.

Results from COPD gene studies have shown that physiologic airway obstruction correlates with both QCT air-trapping indices^26^, ^27^ and QCT-determined emphysema,^26^ with the former showing stronger correlations. Similarly, in asthmatic patients QCT-determined air trapping has been associated with increased disease severity.^28^ Emphysema in asthmatic patients has not been extensively studied. However, a few studies have suggested that emphysema in asthmatic patients is likely secondary to smoking.^29^ In our study we did not find any evidence of emphysema in asthmatic patients because the Perc15 value was comparable with that in healthy control subjects. Perc15 values in the asthma subgroup with severe airflow limitation were similar to those in the COPD subgroup with matched airflow limitation, which might suggest that these asthmatic patients have emphysema. However, high fractal dimension of low-attenuation clusters in the asthma subgroup compared with the COPD subgroup indicate that Perc15 values in this cohort represent air trapping rather than emphysema. Other researchers have found low attenuation on CT scans in asthmatic patients, which is comparable with results seen in patients with emphysema^30^, ^31^ and has been attributed to peribronchial fibrosis or a rupture of dilated bronchial glands rather than the alveolar disruption seen in patients with COPD.^32^ Therefore the fractal dimension of the low-attenuation cluster is an important QCT parameter in differentiating CT low attenuation secondary to emphysema and air trapping.^31^, ^33^

The findings presented here for COPD are consistent with those of previous studies and support the view that airflow limitation and obstruction are due to a combination of small-airway obliteration and emphysema.^34^ We found that changes in proximal airway geometry contribute to postbronchodilator FEV_1_ percent predicted values in the multiple regression model for the whole COPD cohort. This is in keeping with previous studies, which have shown that both emphysema and proximal airway remodeling contribute to the prediction of lung function in patients with COPD.^35^ Proximal airway geometry, particularly airway lumen narrowing, was associated with airflow limitation in asthmatic patients. However, when the asthma subgroup with airflow limitation was assessed, only air trapping was a significant predictor of lung function, suggesting that small-airway disease is particularly important in this group. This might be important for our understanding of disease pathogenesis, monitoring the response to therapy and identification for therapeutic targets. Importantly, emphysema is absent in asthmatic patients with varying degrees of severity and smoking history. Whether the absence of emphysema is a critical distinction between the pathogenesis of asthma and COPD or simply a consequence of the classification of COPD is unclear. Air trapping determined by using QCT was closely related to residual volume/total lung capacity (as a percentage) in both asthmatic patients and patients with COPD, and QCT-determined emphysema was related to KCO percent predicted values in patients with COPD. Even though important differences were observed between asthmatic patients and patients with COPD, there was marked heterogeneity within both disease groups, supporting the view that classification of obstructive airways disease needs to consider multiple dimensions of the disease rather than rely on simple disease labels.

Beyond the associations between QCT and lung function, we explored the relationship between QCT and sputum cell counts or health status. The clinical significance of the weak correlations seen between airway inflammation and Perc15 or low-attenuation cluster of less than −950 HU fractal dimension values in asthmatic patients is uncertain. Proximal airway narrowing in asthmatic patients was associated with an increased blood neutrophil counts. Previous studies have reported similar relationships in asthmatic patients, with airway remodeling and lung function decrease.^21^ There were also weak relationships between proximal airway morphometry and health status in asthmatic patients, with decreased WA and LA values associated with poorer asthma control and health status. In patients with COPD, increased air trapping, but neither proximal wall remodeling nor emphysema, was weakly associated with poorer health status. How closely changes in airway morphometry or densitometry over time or in response to interventions are related to these clinical outcomes needs to be investigated further.

The major limitation of this report is that it is a cross-sectional study, and therefore neither the natural history of disease nor the temporal repeatability of the measures was examined. In previous reports QCT was highly repeatable, and therefore we are confident that the measures are robust, but longitudinal studies are needed to study the dynamic relationships between airway structure and function. Patients with COPD were older than those with asthma and healthy control subjects, and therefore age and disease effects need to be considered. Importantly, in our study population age did not influence the differences in QCT parameters between groups for any of the coprimary QCT outcome measures.

Although this is the largest study to date comparing QCT parameters in asthmatic patients and patients with COPD, to further explore the heterogeneity of QCT in both of these groups, further larger studies that include complex phenotyping are required. The investigation of the relationship between QCT and airway inflammation was limited to sputum cell counts and needs to be extended in larger studies of airway inflammation and remodeling determined from bronchial biopsy specimens. In addition, the effect of disease exacerbations and exposure to pathogens on structure-function relationships needs to be explored further.

In conclusion, proximal airway remodeling and air trapping are QCT features shared by asthmatic patients and patients with COPD compared with healthy control subjects, but emphysema is largely restricted to patients with COPD. In both disease groups air trapping is an independent major determinant for lung function impairment, with an additional important contribution from proximal airway remodeling, particularly in asthmatic patients with mild lung function impairment.

**Clinical implications**

Comprehensive comparisons of QCT parameters between asthmatic patients and patients with COPD and their association with lung function and clinical outcomes might further our understanding of disease pathogenesis, help monitor disease progression, and improve phenotyping of airway disease.

## Figures and Tables

**Table E1:** LA/BSA for segmental airways in asthmatic patients, patients with COPD, and healthy control subjects

	Asthmatic patients	Patients with COPD	Healthy subjects	Significance (*P* value)
RB1 LA/BSA (mm^2^/m^2^)	11.3 (4.04)	10.6 (3.68)	11.8 (3.95)	.25
RB2 LA/BSA (mm^2^/m^2^)	11.4 (3.87)	11.2 (4.26)	12.1 (4.34)	.42
RB3 LA/BSA (mm^2^/m^2^)	15.0 (5.71)	15.3 (5.64)	16.1 (4.58)	.48
RB4 LA/BSA (mm^2^/m^2^)	8.42 (3.36)	8.65 (3.12)	9.17 (3.35)	.37
RB5 LA/BSA (mm^2^/m^2^)	9.38 (3.01)	9.98 (4.41)	10.9 (2.92)	.42* .02† .29‡
RB6 LA/BSA (mm^2^/m^2^)	13.8 (6.51)	15.9 (13.9)	16.0 (6.95)	.19
RB7 LA/BSA (mm^2^/m^2^)	8.86 (4.08)	8.07 (3.07)	9.27 (3.22)	.19
RB8 LA/BSA (mm^2^/m^2^)	10.4 (3.32)	10.3 (3.05)	11.9 (3.06)	.98* .02† **.02‡**
RB9 LA/BSA (mm^2^/m^2^)	8.83 (3.77)	8.20 (3.35)	9.96 (3.25)	.41* .13† **.02‡**
RB10 LA/BSA (mm^2^/m^2^)	11.9 (3.84)	11.84 (4.42)	13.38 (3.71)	.07
LB1 LA/BSA (mm^2^/m^2^)	8.71 (3.11)	8.43 (3.34)	9.92 (3.18)	.81* .07† **.03‡**
LB2 LA/BSA (mm^2^/m^2^)	6.04 (2.67)	6.45 (3.23)	7.66 (4.16)	.63* .007† .10‡
LB3 LA/BSA (mm^2^/m^2^)	13.6 (4.65)	14.4 (5.50)	14.2 (4.35)	.44
LB4 LA/BSA (mm^2^/m^2^)	8.26 (3.25)	8.08 (3.53)	8.77 (3.83)	.54
LB5 LA/BSA (mm^2^/m^2^)	7.58 (2.80)	8.11 (3.09)	8.39 (2.27)	.16
LB6 LA/BSA (mm^2^/m^2^)	17.1 (6.86)	18.6 (7.74)	20.4 (5.63)	.32* .01† .34‡
LB1+2 LA/BSA (mm^2^/m^2^)	13.6 (5.32)	15.3 (7.13)	15.7 (5.69)	.08* .08† .94‡
LB8 LA/BSA (mm^2^/m^2^)	12.3 (3.96)	13.5 (5.67)	15.0 (5.28)	.16* .002† .22‡
LB9 LA/BSA (mm^2^/m^2^)	10.7 (4.46)	10.4 (4.68)	12.7 (4.83)	.87* .03† **.02‡**
LB10 LA/BSA (mm^2^/m^2^)	12.7 (4.40)	12.8 (4.89)	14.1 (4.33)	.17
Right segmental mean LA/BSA (mm^2^/m^2^)	10.9 (2.62)	10.8 (3.57)	12.0 (2.62)	.99* .04† .06‡
Left segmental mean LA/BSA (mm^2^/m^2^)	11.0 (2.79)	11.4 (3.44)	12.4 (3.52)	.66* .02† .18‡

Data are expressed as means (SDs). Intergroup comparison: The *P* value for 1-way ANOVA has been presented unless the ANOVA result was significant (*P* < .05), in which case the *P* value has been presented for Tukey test pairwise comparisons: *asthmatic patients versus patients with COPD, †asthmatic patients versus healthy subjects, and ‡patients with COPD versus healthy subjects.

**Table E2:** %WA for segmental airways for asthmatic patients, patients with COPD, and healthy control subjects

	Asthmatic patients	Patients with COPD	Healthy subjects	Significance (*P* value)
RB1 %WA	62.7 (3.97)	64.0 (3.54)	61.3 (3.39)	.13* .03† **.001‡**
RB2 %WA	61.8 (3.41)	62.3 (3.61)	59.4 (3.65)	.54* 1E-4† **2E-5‡**
RB3 %WA	60.1 (3.67)	60.4 (3.91)	57.9 (3.98)	.79 **.001†** **.001‡**
RB4 %WA	63.3 (3.63)	63.7 (3.17)	61.7 (3.23)	.65* .02† **.005‡**
RB5 %WA	62.8 (3.06)	62.6 (3.33)	60.3 (2.38)	.93* 6E-6† **2E-4‡**
RB6 %WA	61.4 (4.52)	61.4 (4.53)	58.1 (3.95)	1* 1E-4† **.001‡**
RB7 %WA	64.6 (3.71)	66.0 (2.72)	63.3 (3.19)	.02* .07† **2E-4‡**
RB8 %WA	62.8 (3.45)	64.0 (3.16)	60.9 (2.89)	.04* .001† **2E-6‡**
RB9 %WA	63.6 (3.23)	64.4 (2.93)	62.1 (3.56)	.28* .01† **.001‡**
RB10 %WA	61.2 (3.30)	61.6 (3.44)	59.3 (3.43)	.71* .002† **.001‡**
LB1 %WA	63.9 (2.88)	64.3 (2.84)	62.1 (2.68)	.61* .001† **1E-4‡**
LB2 %WA	64.8 (3.04)	64.6 (2.98)	62.6 (4.12)	.94* 4E-4† **.004‡**
LB3 %WA	60.5 (3.92)	60.9 (4.42)	58.9 (3.35)	.8* .04† **.02‡**
LB4 %WA	62.4 (3.65)	63.1 (3.47)	61.1 (3.73)	.53* .08† **.02‡**
LB5 %WA	63.6 (3.21)	63.8 (2.97)	62.1 (2.45)	.87* .007† **.006‡**
LB6 %WA	59.4 (4.56)	59.0 (4.78)	56.1 (3.41)	.85* 4E-5† **.001‡**
LB1+2 %WA	62.1 (4.17)	61.3 (4.54)	59.8 (4.34)	.42* .005† .14‡
LB8 %WA	62.5 (3.60)	62.1 (4.30)	59.6 (4.08)	.73* 4E-5† **.003‡**
LB9 %WA	63.4 (3.65)	63.3 (3.42)	61.4 (3.84)	.96* .002† **.01‡**
LB10 %WA	61.1 (3.88)	61.3 (3.70)	58.7 (3.39)	.87* 3E-4† **4E-4‡**
Right segmental mean %WA	62.50 (2.29)	62.9 (2.45)	60.4 (2.24)	.53* 2E-7† **7E-8‡**
Left segmental mean %WA	62.42 (2.34)	62.4 (2.36)	60.3 (2.28)	.98* 9E-8† **4E-6‡**

Data are expressed as means (SDs). Intergroup comparison: The *P* value for 1-way ANOVA has been presented unless the ANOVA result was significant (*P* < .05), in which case the *P* value has been presented for Tukey test pairwise comparisons: *asthmatic patients versus patients with COPD, †asthmatic patients versus healthy subjects, and ‡patients with COPD versus healthy subjects.

**Table E3:** Correlations between clinical outcomes and QCT parameters for asthmatic patients (upper values) and patients with COPD (lower values)

	RV/TLC	KCO (% predicted)	Sputum eosinophils (%)*	Sputum neutrophils (%)*	Blood eosinophils*	Blood neutrophils*	AQLQ*	ACQ6*	SGRQ total*
Mean LA/BSA (mm^2^/m^2^)	−0.147 −0.171	−0.163 −0.089	0.031 0.121	−0.134 0.036	−0.019 −0.033	−0.183† −0.11	0.113 NA	−0.152† NA	NA −0.034
Mean TA/BSA (mm^2^/m^2^)	−0.115 −0.198	−0.153 −0.084	0.044 0.096	−0.120 0.042	−0.011 −0.033	−0.188† −0.101	0.135 NA	−0.164* NA	NA −0.040
Mean WA/BSA (mm^2^/m^2^)	−0.078 −0.217	−0.153 −0.075	0.064 0.086	−0.119 0.064	−0.003 −0.027	−0.196† −0.28	0.171† NA	−0.179* NA	NA −0.059
Mean %WA	0.237‡ 0.130	0.201† 0.053	−0.003 −0.236	0.094 −0.026	0.037 −0.07	0.128 0.216	−0.049 NA	0.120 NA	NA 0.010
MLD_E/I_	0.481‡ 0.510‡	−0.146 −0.466‡	0.026 −0.170	0.119 0.185	0.081 0.033	0.053 0.135	−0.015 NA	0.030 NA	NA 0.230†
Perc15 (HU)	−0.196† −0.271†	−0.013 0.477‡	−0.239† 0.047	0.084 0.005	−0.007 0.142	0.022 −0.018	−0.028 NA	0.001 NA	NA 0.108
LAC-D−950	−0.162 −0.170	0.083 0.484‡	0.272‡ 0.181	−0.083 0.193	0.156 0.025	−0.074 −0.021	0.006 NA	0.024 NA	NA −0.061

*ACQ6*, Asthma Control Questionnaire (first 6); *AQLQ*, Asthma Quality of Life Questionnaire; *NA*, not applicable; *RV/TLC*, residual volume/total lung capacity; *SGRQ*, St Georges Respiratory Questionnaire.

Pearson correlation coefficient for parametric data are shown. *Otherwise, the Spearman correlation coefficient for nonparametric data is shown.

†*P* < .05 and ‡*P* < .005.

**Table E4:** Correlations between QCT parameters and clinical outcomes in asthmatic patients (n = 70, upper values) and patients with COPD (n = 77, lower values) with FEV_1_ percent predicted values of less than 80%

	Post-BD FEV_1_ (% predicted)	Post-BD FEV/FVC (%)	RV/TLC	KCO (% predicted)	Sputum eosinophils (%)*	Sputum neutrophils*	Blood eosinophils*	Blood neutrophils*	AQLQ*	ACQ6*	SGRQ*
Mean LA/BSA (mm^2^/m^2^)	−0.072 0.158	−0.085 0.043	0.049 −0.106	0.001 −0.120	0.189 0.043	0.093 0.047	0.143 −0.033	0.134 −0.096	0.085 NA	−0.153 NA	NA −0.035
Mean TA/BSA (mm^2^/m^2^)	−0.069 0.164	−0.110 0.044	0.082 −0.145	−0.007 −0.129	0.203 0.021	0.092 0.042	0.152 −0.045	−0.140 −0.087	0.112 NA	−0.170 NA	NA −0.052
Mean WA/BSA (mm^2^/m^2^)	0.070 0.164	−0.137 0.045	0.118 −0.179	−0.007 −0.134	0.215 0.024	0.110 0.052	0.169 −0.044	−0.160 −0.02	0.172 NA	−0.208 NA	NA −0.079
Mean %WA	0.020 −0.161	−0.060 −0.094	0.057 0.046	0.064 0.043	−0.190 −0.153	−0.053 −0.083	−0.101 0.048	−0.103 0.186	0.068 NA	0.004 NA	NA −0.026
MLD_E/I_	−0.455‡ −0.657‡	−0.558‡ −0.714‡	0.611‡ 0.482‡	−0.254 −0.422‡	0.138 −0.135	0.030 0.244	0.066 0.040	−0.158 0.187	0.168 NA	−0.183 NA	NA 0.254†
Perc15 (HU)	0.233 0.458‡	0.493‡ 0.559‡	−0.242 −0.264†	−0.021 0.467‡	−0.042 0.031	0.057 −0.035	0.098 0.132	−0.118 −0.025	−0.157 NA	0.089 NA	NA 0.074
Mean fractal -950	−0.007 −.239†	−0.149 −0.248†	0.048 0.202	−0.019 −0.481‡	0.216 −0.258	0.038 0.289†	0.183 0.077	−0.02 −0.002	0.326‡ NA	−0.340‡ NA	NA 0.133

*ACQ6*, Asthma Control Questionnaire (first 6); *AQLQ*, Asthma Quality of Life Questionnaire; *BD*, bronchodilator; *FVC*, forced vital capacity; *NA*, not applicable; *RV/TLC*, residual volume/total lung capacity; *SGRQ*, St Georges Respiratory Questionnaire.

Pearson correlation coefficient for parametric data is shown. *Otherwise, the Spearman correlation coefficient for nonparametric data is shown.

†*P* < .05 and ‡*P* < .005.

**Table E5:** Multiple regression to determine the strongest independent QCT parameters of FEV_1_ percent predicted in those subjects with FEV_1_ percent predicted values of less than 80%

	Model *R*^*2*^	B	SE	β	Significance (*P* value)
Patients with COPD					
MLD_E/I_	0.473	−241	41.5	−0.567	.001
Perc15 (HU)		0.165	0.073	0.221	.03

**Table E6:** Factor analysis of QCT variables with combined cohort of both asthmatic patients and patients with COPD

	Factors		
1	2	3
Mean LA/BSA (mm^2^/m^2^)	0.99	0.02	−0.05
Mean TA/BSA (mm^2^/m^2^)	0.94	0.02	0.00
Mean WA/BSA (mm^2^/m^2^)	0.98	0.02	−0.02
Mean %WA	−0.78	0.03	0.16
Insp VI−950	−0.06	0.73	0.60
MLD_E/I_	−0.04	0.33	0.87
Mean voxel index change	−0.18	−0.04	0.87
Mean fractal 856	0.15	0.57	0.38
Insp VI−950	−0.08	0.92	0.08
Perc15 (HU)	0.06	−0.94	0.13
Mean fractal 950	0.07	0.75	0.18

The 3 factors accounted for 81% of variation of all variables.

*Insp VI−950*, Inspiratory voxel index less than −950 HU.

**Table E7:** Demographics and both clinical CT and QCT of clusters in a combined cohort of asthmatic patients and patients with COPD

	Cluster 1: asthmatic patients, n = 42; patients with COPD, n = 2	Cluster 2: asthmatic patients, n = 94; patients with COPD, n = 24	Cluster 3: asthmatic patients, n = 25; patients with COPD, n = 47	Significance (*P* value)
Age (y)	49.7 (13.1)	58.0 (12.4)	62.8 (12.5)	6E-7* .03† **.001‡**
Sex§				
Female	73.8%	27.8%	45.8%	**1E-5**
Male	26.2%	72.2%	54.2%	
BMI (kg/m^2^)	29.0 (6.0)	28.9 (5.7)	28.7 (6.3)	.97
Smoking status‖				
Current smoker	4.8%	9.7%	9.3%	**2E-4**
Exsmoker	31%	68.1%	44.9%	
Never smoked	64.3%	22.2%	45.7%	
Pack years (if smoked)	4.8 (10.6)	13.8 (28.0)	34.3 (36.7)	2E-7* 3E-6† .2‡
Severe exacerbations per year	2 (3)	2 (2)	2 (2)	.51
Blood eosinophil count (×10^9^/L)§	0.28 (0.14-0.42)	0.25 (0.15-0.36)	0.23 (0.14-0.29)	.29
Blood neutrophil count (×10^9^/L)§	4.6 (3.4-5.7)	4.4 (3.6-5.7)	4.6 (3.6-5.7)	1
Total sputum cell count (10^6^/g)§	1.77 (0.92-7.84)	2.33 (1.01-5.10)	3.37 (1.23-7.00)	.27
Sputum neutrophils (%)§	44.8 (19.3-73.0)	49.3 (22.0-71.3)	61.5 (17.0-88.5)	.21
Sputum eosinophils (%)§	0.5 (0-2.3)	1.9 (0.3-6.3)	1.0 (0.3-2.5)	.08
Pre-BD FEV_1_ (% predicted)	82.3 (24.4)	77.6 (23.3)	52.2 (21.7)	5E-10* 2E-11† .77‡
Post-BD FEV_1_ (% predicted)	89.2 (21.9)	82.7 (23.5)	57.8 (24.0)	1E-10* 4E-11† .38‡
Pre-BD FEV_1_/FVC ratio (%)	72.7 (9.8)	67.0 (12.0)	50.3 (12.5)	4E-18* 3E-17† .28‡
Post-BD FEV_1_/FVC ratio (%)	76.1 (9.6)	68.5 (11.4)	52.2 (12.7)	4E-21* 2E-17† **.001‡**
BD response	0.19 (0.24)	0.16 (0.21)	0.18 (0.23)	.79
KCO (% predicted)	107 (18.5)	101 (20.3)	79.7 (29.6)	2E-6* 1E-7† .82‡
RV/TLC (%)	37.7 (12)	41.2 (11)	32.7 (12)	3E-8* 2E-8† .40‡
Mean LA/BSA (mm^2^/m^2^)	10.7 (2.94)	11.4 (2.46)	11.0 (3.05)	.29
Mean TA/BSA (mm^2^/m^2^)	27.9 (6.19)	29.6 (5.13)	28.5 (6.16)	.19
Mean WA/BSA (mm^2^/m^2^)	17.2 (3.32)	18.2 (2.78)	17.4 (3.20)	.12
Mean %WA	62.7 (2.21)	62.2 (2.05)	62.6 (2.36)	.3
MLD_E/I_	0.866 (0.056)	0.856 (0.061)	0.910 (0.058)	5E-4* 1E-8† 1.00‡
RVC	−12.93 (11.34)	−23.96 (10.47)	−30.59 (12.22)	4E-16* 9E-20† .71‡
Insp VI−950	4.6 (2.0)	13.0 (3.2)	26.6 (6.13)	1E-72* 6E-57† **1E-22‡**
Exp VI−856	13.3 (8.3)	21.7 (12.6)	45.8 (18.8)	1E-22* 5E-20† **.004‡**
CTLV_E/I_	0.65 (0.09)	0.58 (0.11)	0.66 (0.13)	1.00* 4E-5† **.004‡**
Perc15 (HU)	−905 (14.7)	−943 (8.7)	−972 (11.1)	5E-86* 1E-43† **1E-51‡**
LAC-D−950	−1.92 (0.20)	−1.84 (0.12)	−1.80 (0.13)	.22
Pi10 (mm^2^)	14.9 (1.28)	15.0 (1.41)	14.8 (1.28)	.73
Po20 %WA	56.2 (2.37)	56.0 (2.34)	56.0 (2.83)	.88
%WA (no. [%])§#	6 (14.3)	15 (12.7)	11 (15.3)	.88
MLD_E/I_ (no. [%])§#	2 (4.8)	8 (6.8)	20 (27.8)	**8E-5**
Perc15 (no. [%])‖	0 (0)	0 (0)	31 (43.1)	**6E-19**

*BD*, Bronchodilator; *BMI*, body mass index; *CTLV*_*E/I*_, computed tomographic lung volume expiratory/inspiratory ratio; *Exp VI−856*, expiratory voxel index less than −856 HU; *FVC*, forced vital capacity; *Insp VI−950*, inspiratory voxel index less than −950 HU; *Pi10*, wall area of theoretical airway with an internal perimeter of 10 mm; *Po20 %WA*, percentage wall area of a theoretical airway with an external perimeter of 20 mm; *RVC*, relative voxel change; *RV/TLC*, residual volume/total lung capacity.

Intergroup comparison, parametric (nonparametric) data: The *P* value for 1-way ANOVA (Kruskal-Wallis test) has been presented unless the ANOVA (Kruskal-Wallis test) result was significant (*P* < .05), in which case the *P* value has been presented for the Tukey (Dunn) test pairwise comparisons: *asthmatic patients versus patients with COPD, †asthmatic patients versus healthy subjects, and ‡patients with COPD versus healthy subjects. Differences in proportions were tested by using the χ^2^ test. Data are expressed as means (SDs), §medians (interquartile ranges), or §proportions. #Greater than 2 SDs of healthy control subjects. ‖Less than 2 SDs of healthy control subjects.

**Table I:** Clinical characteristics of all asthmatic patients, patients with COPD, and healthy control subjects

	Asthmatic patients (n = 171)	Patients with COPD (n = 81)	Healthy subjects (n = 49)	Significance (*P* value)
Age (y)	53 (12.8)	69 (8.16)	57 (13.3)	5E-9* .07† 2E-7‡
Sex‖				
Female	51%	33%	39%	.03
Male	49%	67%	61%	
BMI (kg/m^2^)	30 (6)	28 (5)	29 (5)	.02* .98† .07‡
Smoking status‖				
Current smoker	4%	20%	4%	**7E-17**
Exsmoker	34%	80%	45%	
Never smoked	62%	0%	51%	
Pack years (if smoked)	12.3 (10.6)	50.5 (31.2)	11.7 (9.20)	5E-9* .99† **5E-9‡**
Severe exacerbations per year	2.20 (2.58)	2.18 (2.20)	0	1*
AQLQ	4.97 (1.33)	NA	NA	
ACQ6	1.81 (1.15)	NA	NA	
SGRQ total	NA	49.8 (19.1)	NA	
GOLD/GINA % per group 1/2/3/4 (5)	9/5/19/40 (27)	5/55/29/11	NA	
Total IgE (kU/L)	490 (1785)	ND	83.6 (217)	.13†
Blood eosinophil count (×10^9^/L)§	0.26 (0.15-0.39)	0.22 (0.14-0.29)	0.13 (0.1-0.2)	.08* 1E-8† **.03‡**
Blood neutrophil count (×10^9^/L)§	4.42 (3.43-5.77)	4.56 (3.7-5.47)	3.74 (3.16-4.46)	1* .01† **.005‡**
Total sputum cell count (10^6^/g)§	2.25 (1.13-5.44)	3.92 (1.32-8.46)	1.64 (0.49-5.7)	.24* .51† **.04‡**
Sputum (% neutrophils)§	51.8 (35.3-73)	75.5 (39.8-89.8)	75.1 (48.5-90.3)	.007* .006† 1‡
Sputum (% eosinophils)§	2.25 (0.5-8.5)	0.75 (0.25-2)	0.25 (0-0.75)	1E-8* 1E-8† .1‡
Pre-BD FEV_1_ (% predicted)	78.2 (25.2)	50.5 (17.6)	111 (17.2)	5E-9* 5E-9† **5E-9‡**
Post-BD FEV_1_ (% predicted)	85.3 (24.3)	53.7 (17.2)	113 (18.4)	5E-9* 5E-9† **5E-9‡**
Pre-BD FEV_1_/FVC ratio (%)	68.5 (13.3)	50.6 (10.6)	78.5 (5.55)	5E-9* 9E-7† **5E-9‡**
Post-BD FEV_1_/FVC ratio (%)	70.7 (12.0)	51.7 (10.2)	78.5 (12.6)	5E-9* 1.5E-4† **5E-9‡**
BD response (%)	11.3 (15.1)	8.12 (9.56)	1.78 (4.36)	.17* 2E-5† **.019‡**
KCO (% predicted)	107 (18.4)	74.8 (25.6)	98.9 (13.5)	5E-9* .08† **3E-8‡**
RV/TLC (%)	39.7 (12)	55.1 (12)	34.5 (9)	5E-9* .04† **5E-9‡**

*ACQ6*, Asthma Control Questionnaire (first 6); *AQLQ*, Asthma Quality of Life Questionnaire; *BD*, bronchodilator; *BMI*, body mass index; *FVC*, forced vital capacity; *GINA*, Global Initiative for Asthma; *GOLD*, Global Initiative for Chronic Obstructive Lung Disease; *NA*, not applicable; *ND*, not done; *RV/TLC*, residual volume/total lung capacity; *SGRQ*, St Georges Respiratory Questionnaire.

Intergroup comparison, parametric (nonparametric) data: The *P* value for 1-way ANOVA (Kruskal-Wallis test) has been presented unless the ANOVA (Kruskal-Wallis test) result was significant (*P* < .05), in which case the *P* value has been presented for the Tukey (Dunn) test pairwise comparisons: *asthmatic patients versus patients with COPD, †asthmatic patients versus healthy subjects, and ‡patients with COPD versus healthy subjects. Differences in proportions were tested by using the χ^2^ test.

Data are expressed as means (SDs), §medians (interquartile ranges), or ‖proportions.

**Table II:** Airway morphometry and lung densitometry of asthmatic patients, patients with COPD, and healthy control subjects

	Asthmatic patients (n = 171)	Patients with COPD (n = 81)	Healthy subjects (n = 49)	Significance (*P* value)
Mean LA/BSA (mm^2^/m^2^)	11.0 (2.58)	11.3 (3.02)	12.3 (2.75)	.67* .006† .08‡
Mean TA/BSA (mm^2^/m^2^)	28.5 (5.32)	29.3 (6.20)	30.5 (5.40)	.09
Mean WA/BSA (mm^2^/m^2^)	17.5 (2.84)	18.1 (3.31)	18.1 (2.76)	.29
Mean %WA	62.5 (2.19)	62.7 (2.26)	60.3 (2.17)	.79* 3E-8† **4E-8‡**
MLD_E/I_	0.852 (0.061)	0.922 (0.037)	0.816 (0.066)	5E-9* 5E-4† **5E-9‡**
RVC	−29.3 (12.4)	−12.2 (9.36)	−36.8 (10.2)	5E-9* 3E-4† **5E-9‡**
Insp VI−950	12.17	23.32	11.40	5E-9* .79 **5E-9‡**
Exp VI−856	20.27	47.57	14.81	5E-9* <.05† **5E-9‡**
CTLV_E/I_	0.58 (0.13)	0.67 (0.18)	0.51 (0.12)	5E-9* .009† **8E-9‡**
Perc15 (HU)	−937 (22.7)	−964 (19.62)	−937 (17.07)	5E-9* 1† **5E-9‡**
LAC-D−950	1.96 (0.104)	1.810 (0.132)	1.989 (0.107)	6E-9* .26† **1E-8‡**
Pi10 WA (mm^2^)	15.1 (1.42)	15.0 (1.46)	14.4 (1.10)	.89* .011† .06‡
Po20 %WA	56.1 (2.57)	56.4 (2.97)	54.6 (1.71)	.7* .001† **2E-4‡**
%WA (no. [%] above)§	27 (15.8%)	13 (7.60%)	NA	1.0
MLD_E/I_ (no. [%] above)§	8 (4.68%)	22 (27.16%)	NA	**1E-8**
Perc15 (no. [%] below)‖	7 (4.09%)	26 (32.1%)	NA	**1E-8**

*CTLV*_*E/I*_, Computed tomographic lung volume expiratory/inspiratory ratio; *Exp VI−856*, expiratory voxel index less than −856 HU; *Insp VI−950*, inspiratory voxel index less than −950 HU; *Pi10*, Wall area of theoretical airway with an internal perimeter of 10 mm; *Po20 %WA*, Percentage wall area of a theoretical airway with an external perimeter of 20 mm; *RVC*, Relative voxel change.

Intergroup comparison: The *P* value for 1-way ANOVA has been presented unless the ANOVA result was significant (*P* < .05), in which case the *P* value has been presented for Tukey test pairwise comparisons: *asthmatic patients versus patients with COPD, †asthmatic patients versus healthy subjects, and ‡patients with COPD versus healthy subjects.

Data are expressed as means (SDs), §greater than 2 SDs of healthy control subjects, and ‖less than 2 SDs of healthy control subjects.

**Table III:** Correlations between clinical outcomes and QCT parameters

	Postbronchodilator FEV_1_ (% predicted), asthmatic patients	Postbronchodilator FEV_1_ (% predicted), patients with COPD	Postbronchodilator FEV_1_/FVC (%), asthmatic patients	Postbronchodilator FEV_1_/FVC (%), patients with COPD
Mean LA/BSA (mm^2^/m^2^)	0.324†	0.241*	0.218†	0.082
Mean TA/BSA (mm^2^/m^2^)	0.287†	0.238*	0.171*	0.084
Mean WA/BSA (mm^2^/m^2^)	0.247†	0.226*	0.126	0.083
Mean %WA	−0.417†	−0.248*	−0.343†	−0.121
MLD_E/I_	−0.303†	−0.697†	−0.402†	−0.729†
Perc15 (HU)	0.178*	0.434†	0.408†	0.554†
LAC-D−950	0.190*	0.180	0.234†	0.245*

*FVC*, Forced vital capacity.

Pearson correlation coefficient: *P < 0.05 and †*P* < .005.

**Table IV:** Multiple regression to determine the strongest independent QCT parameters of postbronchodilator FEV_1_ percent predicted (ie, the dependent variable)

	Model *R*^*2*^	B	SE	β	Significance (*P* value)
Asthmatic patient					
%WA	0.254	−3.771	0.778	−0.344	**3E-6**
MLD_E/I_		−108.021	28.283	−0.271	**2E-4**
Perc15 (HU)		0.190	0.074	.0181	**.01**
Patients with COPD					
%WA	0.542	−1.447	0.644	−0.185	**.03**
MLD_E/I_		−283.191	42.260	−0.607	**5E-9**
Perc15 (HU)		0.151	0.079	0.173	.06

## Methods

### CT

Volumetric whole lung scans were obtained by using a Siemens Sensation 16 scanner (16 × 0.75–mm collimation, 1.5-mm pitch, 120 kVp, 40 mAs, 0.5-second rotation time, and scanning field of view of 500 mm). Scans were obtained at full inspiration (near total lung capacity) and at the end of expiration (near functional residual capacity). All subjects were coached in breath-holding techniques and practiced breath holding immediately before scanning. All asthmatic patients and patients with COPD were scanned within 60 minutes of receiving 400 μg of salbutamol through a spacer or 2.5 mg of nebulized salbutamol. Images were reconstructed with a slice thickness of 0.75 mm at a 0.5-mm interval by using B35f kernal. Postprocessing was performed with the fully automated software Apollo (VIDA Diagnostics).

QCT parameters obtained included the following: morphometry measured in square millimeters, LA, TA, WA (*TA*−*LA*), and $\%\text{WA}\left(100\times \left(\frac{\left(TA-LA\right)}{TA}\right)\right)$. Air-trapping measures were MLD_E/I_ measured in Hounsfield units and relative voxel change *Exp*((*VI*−856) − (*VI*−950)) − *Insp*((*VI*−856) − (*VI*−950)). Emphysema was quantified by using Perc15. Fractal dimensions of the low-attenuation clusters on inspiratory (LAC-D−950) and expiratory (low-attenuation clusters less than −856 HU fractal dimension value) scans were also measured. All morphometric measures were corrected for BSA in square millimeters per square meter $\left(\sqrt{\frac{height\left(cm\right)\times weight\left(kg\right)}{3600}}\right)$. Density measures were corrected to account for scanner variability over time, as previously published.^E1^ A representative example of an inspiratory and expiratory scan, airway reconstruction from the inspiratory scan, and densitometric maps from both the inspiratory and expiratory scans are as shown Fig E1.

### Interobserver repeatability of coprimary QCT parameters

Interobserver repeatability was assessed (Cronbach α intraclass correlation [ICC]) between 2 observers (RH and SG) in 76 subjects for Perc15 (ICC, 0.996; *P* < .001), MLD at inspiration (ICC, 0.997; *P* < .001) and expiration (ICC, 0.997; *P* < .001), RB1 LA (ICC, 0.873; *P* < .001), and TA (ICC, 0.873; *P* < .001).

### Factor and cluster in the COPD and severe asthma cohorts

We undertook *de novo* cluster analysis on the patients with COPD and asthmatic patients together by using the same methodology carried out in Gupta et al^E1^ but using the QCT variables as follows: (1) mean LA/BSA; (2) mean WA/BSA; (3) mean total area (TA)/BSA; (4) mean %WA; (5) expiratory voxel index −856; (6) MLD_E/I_ ratio; (7) voxel index change of percent voxels between −950 HU and −856 HU on paired inspiratory and expiratory CT scan; (8) expiratory fractal dimension of low attenuation cluster at threshold of −856 HU; (9) inspiratory voxel index −950; (10) Perc15; and (11) inspiratory fractal dimension of low attenuation cluster at threshold of −950 HU.

QCT variables listed were first used in a factor analysis, with 3 factors being found, allowing for 81% of the variation. The Kaiser criteria to determine the number of factors were used while picking all factors with an eigenvalue of greater than 1. Varimax rotation was also used to determine the best clinical interpretable factors, as shown in Table E6. The highest loading variables on each factor were taken forward into a cluster analysis. These were mean LA adjusted for BSA, Perc15, and MLD_E/I_ values.

First hierarchic cluster analysis was applied to determine the number of clusters that best fit the data. Then the number of clusters determined (3 in this case, see Fig E3) was inputted into a k-means cluster analysis to determine cluster membership for each patient. Cluster demographics and comparisons are as shown in Table E7.
